# Efficacy and safety of erenumab in women with a history of menstrual migraine

**DOI:** 10.1186/s10194-020-01167-6

**Published:** 2020-08-03

**Authors:** Jelena M. Pavlovic, Koen Paemeleire, Hartmut Göbel, Jo Bonner, Alan Rapoport, Risa Kagan, Feng Zhang, Hernan Picard, Daniel D. Mikol

**Affiliations:** 1 Department of Neurology, Montefiore Headache Center, 1300 Morris Park Avenue, Van Etten 3C9, Bronx, NY 10461 USA; 2 Albert Einstein College of Medicine, Bronx, NY USA; 3 Ghent University Hospital, Ghent, Belgium; 4 Kiel Migraine and Headache Center, Kiel, Germany; 5 Mercy Clinic Neurology, St Louis, MO USA; 6 The David Geffen School of Medicine at UCLA, Los Angeles, CA USA; 7 Department of Obstetrics, Gynecology and Reproductive Sciences, University of California, San Francisco, CA USA; 8 Sutter East Bay Medical Foundation, Berkeley, CA USA; 9 Amgen Inc., Thousand Oaks, CA USA

**Keywords:** Erenumab, Headache, Episodic migraine, Migraine prevention, Pure menstrual migraine, Menstrually related migraine, Perimenstrual attacks

## Abstract

**Background:**

We performed a post hoc, subgroup analysis of a phase 3, randomized, double-blind, placebo-controlled study of erenumab for prevention of episodic migraine (STRIVE) to determine the efficacy and safety of erenumab in women with self-reported menstrual migraine.

**Methods:**

Patients received placebo, erenumab 70 mg, or erenumab 140 mg subcutaneously once monthly during the 6-month double-blind treatment phase of STRIVE. Women who reported history of menstrual migraine and who were ≤ 50 years old were included in the analysis. Endpoints were change from baseline in monthly migraine days (MMD) and monthly acute migraine-specific medication days (MSMD; among patients who took acute migraine-specific medications at baseline), proportion of patients achieving ≥ 50% reduction from baseline in MMD, and incidence of adverse events.

**Results:**

Among 814 women enrolled in STRIVE, 232 (28.5%) reported a history of menstrual migraine and were ≤ 50 years old. Of the 232 patients, 214 (92%) had a baseline MMD > 5, suggesting a high proportion of women with attacks outside of the 5-day perimenstrual window (2 days before and 3 days after the start of menstruation). Information on “migraine days” includes (and does not discriminate between) perimenstrual and intermenstrual migraine attacks. Between-group differences from placebo over months 4–6 for erenumab 70 mg and 140 mg were − 1.8 (*P* = 0.001) and − 2.1 (*P* < 0.001) days for MMD and − 1.6 (*P* = 0.002) and − 2.4 (*P* < 0.001) days for acute MSMD, respectively. The odds of having a ≥ 50% reduction from baseline in MMD over months 4–6 were 2.2 (*P* = 0.024) and 2.8 (*P* = 0.002) times greater for erenumab 70 mg and 140 mg, respectively, than for placebo. Erenumab had an overall safety profile comparable to placebo.

**Conclusion:**

Data from this subgroup analysis of women with menstrual migraine are consistent with data from the overall STRIVE episodic migraine population, supporting the efficacy and safety of erenumab in women who experience menstrual migraine.

Trial registration: ClinicalTrials.gov, NCT02456740. Registered 28 May 2015.

## Background

More than 50% of women self-report an association between migraine and menses [1]. These perimenstrual attacks are commonly referred to as menstrual migraine if they occur within a 5-day window (2 days prior to menstruation and the first 3 days of menstruation). Menstrual migraine attacks are particularly burdensome, as they tend to be of longer duration [2–7] and are more severe and disabling [2, 3, 5, 7–9] than non-perimenstrual attacks. Furthermore, perimenstrual attacks are less responsive to acute therapy, making them difficult to treat [2, 4, 5].

Women who experience migraine attacks with the majority of their menstrual periods (defined by The International Classification of Headache Disorders [ICHD] 3 beta [10] as at least two of three periods) are classified into either pure menstrual migraine (PMM; attacks that occur only during the 5-day perimenstrual window) or menstrually related migraine (MRM; attacks that occur during the 5-day perimenstrual window and at other times of the cycle). PMM is a rare condition that affects approximately 5%–8% of women with migraine [9, 11, 12], with most women self-reporting MRM [4]; however, the percentage varies widely depending on the study populations and diagnostic criteria used [2, 4, 11, 13].

There are no approved, specific preventive treatments for menstrual migraine. It has been proposed that women who do not respond to acute treatment options may be eligible to receive either short-term or long-term preventive treatments [14]. Several medications, including triptans, nonsteroidal anti-inflammatory drugs, cyclooxygenase-2 inhibitors, and estrogen supplementation, have been investigated for short-term prevention of menstrual migraine; however, these agents may delay rather than prevent attacks [14]. Long-term preventive treatment for menstrual migraine has been investigated with topiramate, which reduced the frequency but not the severity or duration of perimenstrual attacks [15]. Continuous use of hormonal contraceptives can reduce the severity and duration of migraine attacks [14, 16–18]. Although hormonal contraceptives containing estrogens are considered a viable treatment option for women with menstrual migraine [19], evidence of their effectiveness is limited [20], and they may be contraindicated because of their association with increased risk of stroke [21–24]. According to the current guidelines, exogenous estrogens are contraindicated in all women with migraine with aura and in women with migraine without aura who are smokers and/or older than 35 years of age [25–27]; estrogen-containing options are therefore often not available to most women with migraine aged 35 or older [25, 26].

The challenges associated with the treatment of menstrual migraine emphasize the need for novel, nonhormonal, long-term preventive treatments. Erenumab is a fully human monoclonal antibody that selectively targets and blocks the canonical calcitonin gene-related peptide (CGRP) receptor [28]. In the 6-month double-blind treatment phase of the STRIVE trial of patients with episodic migraine, erenumab at 70 mg or 140 mg once monthly significantly reduced the number of monthly migraine days (MMD) and monthly acute migraine-specific medication days (MSMD) and increased the odds of achieving ≥50% reduction from baseline in MMD [29]. Given the burden and challenges in the treatment of menstrual migraine, we performed a post hoc subgroup analysis of STRIVE to determine the efficacy and safety of erenumab in women with self-reported menstrual migraine.

## Methods

### Study design and patients

STRIVE (ClinicalTrials.gov, NCT02456740) was a phase 3, randomized, double-blind, placebo-controlled study of erenumab in patients with episodic migraine [29]. In brief, the study consisted of a 7-week screening phase (including 4 weeks of baseline), a 6-month double-blind treatment phase, a 7-month dose-blinded active treatment phase, and a 3-month safety follow-up phase. Randomization was stratified by region (North America vs other) and prior preventive medication status (naïve vs prior use vs concomitant use). Placebo and erenumab 70 mg and 140 mg were administered subcutaneously once every month during the double-blind treatment phase; erenumab 70 mg or 140 mg were administered during the 28-week active treatment phase.

Eligible patients were 18–65 years old with a history of migraine with or without aura (based on medical records and/or self-reported) for at least 12 months before screening. Episodic migraine was defined as an average of 4–14 migraine days per month with fewer than 15 headache days per month (in accordance with ICHD-3) during the 3 months before screening and during the 4-week baseline phase of the study. One concomitant migraine-preventive medication was allowed following a protocol amendment that was introduced late during the enrollment period. Patients were excluded if they had no therapeutic response to > 2 migraine-preventive treatment categories, defined as no reduction in headache frequency, duration, or severity after administration of the medication for at least 6 weeks at the generally accepted therapeutic dose(s) based on the investigator’s assessment.

The study protocol was approved by the ethics committee or institutional review board at each clinical site, and all patients provided signed informed consent before the start of any study-related procedures. The study was conducted in accordance with the International Council for Harmonisation Good Clinical Practice Guidelines and conforms to the provisions of the Declaration of Helsinki.

### Menstrual migraine subgroups

Women were asked if they had migraine attacks that occurred within a 5-day window (2 days prior to menstruation and the first 3 days of menstruation) in at least 2 out of the last 3 menstrual cycles prior to screening in accordance with the criteria for menstrual migraine diagnosis [10]. In industrialized countries, the average age for onset of perimenopause is 47.5 years and is influenced by several demographic, lifestyle, and biologic factors [30]. Based on this, for the current subgroup analysis, we included menstruating women aged ≤ 50 years with a self-reported history of menstrual migraine attacks. Since the data collected did not allow us to distinguish between women who had only menstrual attacks (PMM) and those who had both menstrual and non-menstrual attacks (MRM), both categories are included under the label “menstrual migraine” in our analyses.

### Endpoints

Efficacy endpoints were change from baseline in mean MMD, change from baseline in mean monthly acute MSMD among patients who took acute migraine-specific medications at baseline, and the proportion of patients achieving a ≥ 50% reduction from baseline in MMD (proportion of responders). Efficacy was assessed for each monthly interval from data collected daily using the patients’ electronic diaries; the primary time point of assessment in the study was the average monthly effect over months 4–6. Analysis of migraine frequency–related endpoints includes all (both perimenstrual and intermenstrual) migraine days.

Safety was monitored throughout the study, and adverse events were coded according to the Medical Dictionary for Regulatory Activities version 19.0.

### Statistical analysis

Change from baseline in MMD and monthly acute MSMD was analyzed using a generalized linear mixed effects model, which included treatment, visit, treatment by visit interaction, stratification factors (North America/other and naïve/prior use/concomitant use), and baseline value as covariates and assumed a first-order autoregressive covariance structure; missing data were not imputed. The proportion of responders was analyzed using a stratified Cochran-Mantel-Haenszel test after imputation of missing data as nonresponse. *P* values for the between-group differences (erenumab 70 mg and 140 mg vs placebo) are nominal *P* values without multiplicity adjustment. Statistical significance was determined based on the comparison of the nominal *P* values with a significance level of 0.05.

## Results

### Patient characteristics

Among 814 women enrolled in STRIVE, 232 (28.5%) self-reported a history of menstrual migraine and were ≤ 50 years old. Baseline characteristics were fairly balanced among the treatment groups (Table 1).

**Table 1:** Baseline characteristics

	History of Menstrual Migraine			
	Placebo ***N*** = 83	Erenumab 70 mg ***N*** = 68	Erenumab 140 mg ***N*** = 81	All Patients ***N*** = 232
Age, years, median (range)	37 (20–49)	38 (21–50)	37 (19–50)	37 (19–50)
Race, n (%)				
White	71 (86)	61 (90)	77 (95)	209 (90)
Black or African American	3 (4)	5 (7)	2 (3)	10 (4)
Asian	4 (5)	0 (0)	2 (3)	6 (3)
Other	5 (6)	2 (3)	0 (0)	7 (3)
Migraine with aura,^a^ n (%)	48 (58)	35 (52)	38 (47)	121 (52)
Migraine without aura,^a^ n (%)	71 (86)	61 (90)	72 (89)	204 (88)
Received hormonal contraception, n (%)	20 (24)	18 (26)	27 (33)	65 (28)
Treatment with migraine-preventive medication, n (%)				
Naïve	50 (60)	37 (54)	48 (59)	135 (58)
Prior and/or concomitant use	33 (40)	31 (46)	33 (41)	97 (42)
Baseline phase (4 weeks)				
Monthly migraine days, mean (SD)	8.6 (2.8)	8.3 (2.4)	8.4 (2.4)	8.4 (2.5)
Monthly acute migraine-specific medication days, mean (SD)	3.2 (3.5)	3.1 (3.1)	3.7 (3.6)	3.3 (3.4)
Acute migraine-specific medication use, n (%)	48 (58)	38 (56)	51 (63)	137 (59)

^a^Based on self-report; categories are not mutually exclusive

*Abbreviations*: *SD* standard deviation

Of the 232 women with menstrual migraine, 65 (28%) were taking oral contraceptives/hormone therapy during the study: 18 (26%) in the erenumab 70 mg group, 27 (33%) in the erenumab 140 mg group, and 20 (24%) in the placebo group.

### Efficacy

#### Change from baseline in mean monthly migraine days

**Fig. 1:**
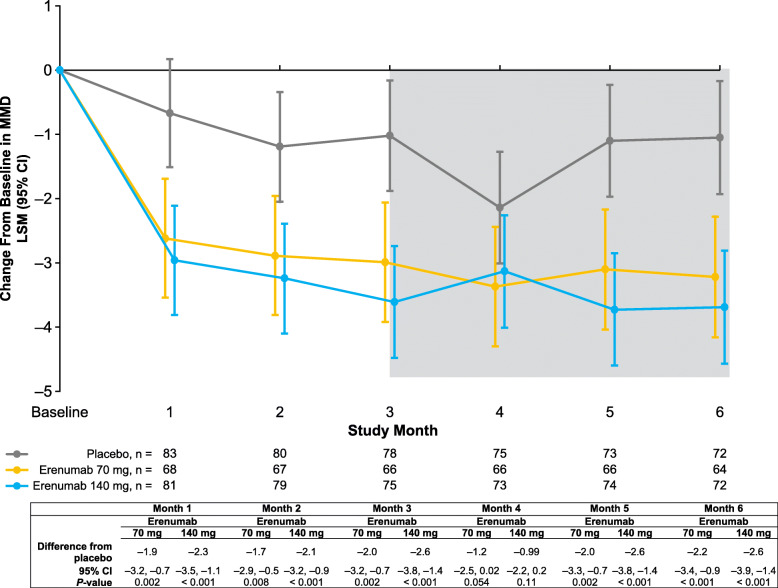
Change from baseline in MMD. Data are shown as LSM with 95% CIs. The gray shaded area represents months 4–6. Abbreviations: CI, confidence interval; LSM, least squares mean; MMD, monthly migraine days

During the study, both doses of erenumab resulted in statistically significantly greater reductions vs placebo in MMD as early as month 1 (Fig. 1). The mean MMD reduction over months 4–6 was − 1.4, − 3.2, and − 3.5 days in the placebo, erenumab 70 mg, and erenumab 140 mg groups, respectively (Table 2). Differences from placebo were statistically significant: –1.8 (*P* = 0.001) and − 2.1 (*P* < 0.001) days for the erenumab 70 mg and 140 mg groups, respectively (Table 2).

**Table 2:** Efficacy over months 4–6 of the double-blind treatment phase

	Placebo ***N*** = 83	Erenumab 70 mg ***N*** = 68	Erenumab 140 mg ***N*** = 81
**Migraine days per month**			
N	83	68	81
Change from baseline, LSM (95% CI)	− 1.4 (− 2.2, − 0.7)	−3.2 (− 4.0, − 2.4)	−3.5 (− 4.3, − 2.8)
Difference from placebo, LSM (95% CI)		−1.8 (− 2.9, − 0.7) *P* = 0.001	−2.1 (− 3.1, − 1.1) *P* < 0.001
**Acute MSMD per month among patients taking acute migraine-specific medications at baseline**			
N	48	38	51
Change from baseline, LSM (95% CI)	−0.4 (−1.1, 0.3)	−2.0 (− 2.8, − 1.2)	−2.8 (− 3.5, − 2.1)
Difference from placebo, LSM (95% CI)		−1.6 (− 2.6, − 0.6) *P* = 0.002	−2.4 (− 3.4, − 1.4) *P* < 0.001
**Patients with ≥ 50% reduction from baseline in migraine days per month (≥ 50% response)**			
N	83	68	81
n (%)	21 (25.3)	29 (42.6)	40 (49.4)
OR^a^ (95% CI)		2.2 (1.1, 4.4) *P* = 0.024	2.8 (1.5, 5.5) *P =* 0.002

The analysis included randomized patients who received ≥ 1 dose of investigational product and had ≥ 1 postbaseline measurement during the double-blind treatment phase. Change from baseline in MMD and monthly acute MSMD was analyzed using a generalized linear mixed effects model, which included treatment, visit, treatment by visit interaction, stratification factors (North America/other and naïve/prior use/concomitant use), and baseline value as covariates and assumed a first-order autoregressive covariance structure; missing data were not imputed. The proportion of responders was analyzed using a stratified Cochran-Mantel-Haenszel test after imputation of missing data as nonresponse

*Abbreviations*: *CI* confidence interval, *LSM* least squares mean, *MSMD* migraine-specific medication days, *OR* odds ratio

^a^The common ORs and *P* values were obtained from a Cochran-Mantel-Haenszel test, stratified by prior/current treatment with migraine-preventive medication and region

An analysis of MMD was performed for patients who were receiving exogenous hormones for contraception versus those who were not receiving exogeneous hormones (Table 3). Overall, the subgroup of patients receiving exogenous hormones had similar efficacy results compared to the total population with a history of menstrual migraine.

**Table 3:** Change From Baseline in Mean Monthly Migraine Days by Hormonal Contraception Status

	Received Hormonal Contraception			Did Not Receive Hormonal Contraception		
	Placebo *N* = 20	Erenumab 70 mg *N* = 18	Erenumab 140 mg *N* = 27	Placebo *N* = 63	Erenumab 70 mg *N* = 50	Erenumab 140 mg *N* = 54
Monthly migraine days at baseline, mean (SD)	9.5 (3.0)	8.5 (2.7)	8.9 (2.2)	8.3 (2.8)	8.2 (2.3)	8.2 (2.4)
Change from baseline over months 4–6, LSM (95% CI)	−1.6 (− 3.4, 0.25)	− 2.9 (− 4.8, − 1.0)	− 3.9 (− 5.5, − 2.4)	− 1.4 (− 2.2, − 0.6)	−3.3 (− 4.2, − 2.4)	−3.4 (− 4.2, − 2.5)
Difference from placebo		−1.3 (− 3.9, 1.2) *P* = 0.3	− 2.4 (− 4.7, − 0.1) *P* = 0.045		−2.0 (− 3.1, − 0.8) *P* = 0.001	−2.0 (− 3.1, − 0.8) *P* < 0.001
Treatment by subgroup interaction *P* value over months 4–6	0.76					

The analysis included randomized patients who received ≥ 1 dose of investigational product and had ≥ 1 postbaseline measurement during the double-blind treatment phase. Change from baseline in MMD and monthly acute MSMD was analyzed using a generalized linear mixed effects model, which included treatment, visit, treatment by visit interaction, stratification factors (North America/other and naïve/prior use/concomitant use), and baseline value as covariates and assumed a first-order autoregressive covariance structure; missing data were not imputed. *P* values for pairwise comparisons are nominal *P* values without multiplicity adjustment

*Abbreviations*: *CI* confidence interval, *LSM* least squares mean

#### Change from baseline in monthly acute migraine-specific medication days

**Fig. 2:**
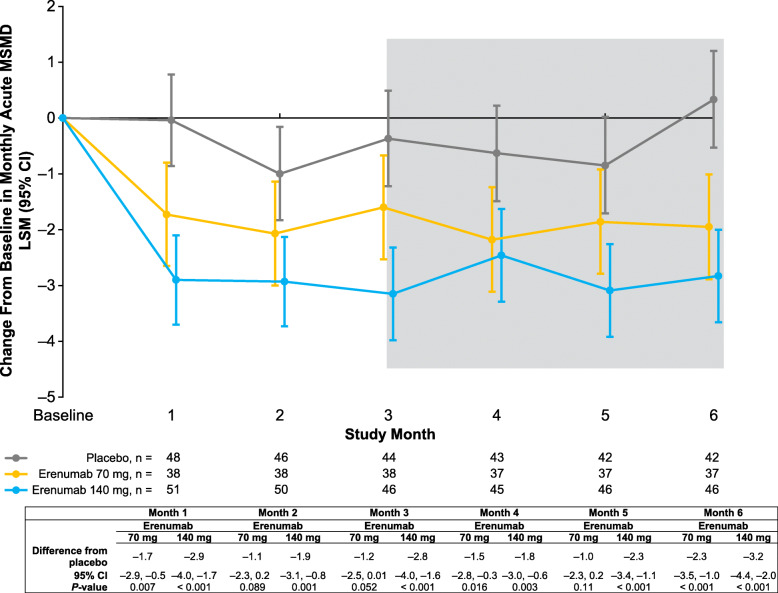
Change from baseline in monthly acute MSMD among patients with a self-reported history of menstrual migraine who took migraine-specific medications at baseline. Data are shown as LSM with 95% CIs. The gray shaded area represents months 4–6. Abbreviations: CI, confidence interval; LSM, least squares mean; MSMD, migraine-specific medication days

In the subgroup of patients who were taking acute migraine-specific medications at baseline, erenumab 70 mg and 140 mg vs placebo resulted in greater reductions in monthly acute MSMD starting at month 1; reductions were statistically significant at every month for the 140-mg dose group (Fig. 2). The mean reduction in monthly acute MSMD over months 4–6 was 0.4, 2.0, and 2.8 days in the placebo, erenumab 70 mg, and erenumab 140 mg groups, respectively (Table 2). Differences from placebo were statistically significant: –1.6 (*P* = 0.002) and − 2.4 (*P* < 0.001) days for the erenumab 70 mg and 140 mg groups, respectively (Table 2).

#### Proportion of patients achieving ≥50% reduction from baseline in monthly migraine days

**Fig. 3:**
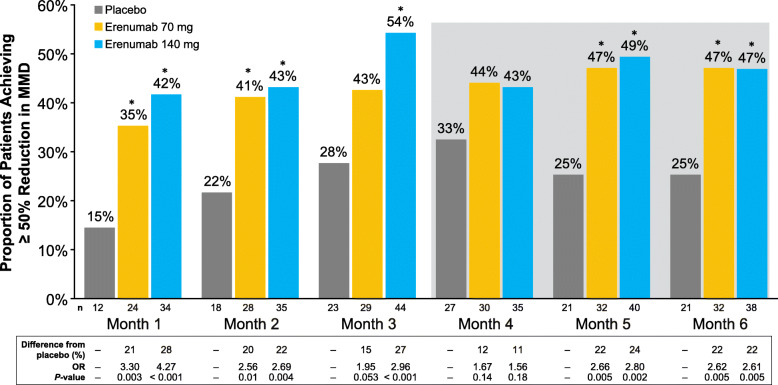
Proportion of patients achieving ≥ 50% reduction from baseline in MMD. Data are shown as percentages. The gray shaded area represents months 4–6. *Statistically significantly different from placebo. Abbreviations: MMD, monthly migraine days; OR, odds ratio

Both doses of erenumab vs placebo resulted in a significantly higher proportion of patients achieving at least a 50% response at each time point except month 4 (Fig. 3). A ≥ 50% response over months 4–6 was achieved by 25.3%, 42.6%, and 49.4% of patients who received placebo, erenumab 70 mg, and erenumab 140 mg, respectively. The odds of having a ≥ 50% response over months 4–6 were 2.2 (*P* = 0.024) and 2.8 (*P* = 0.002) times greater for the erenumab 70 and 140 mg groups, respectively, than for the placebo group (Table 2).

### Safety

Erenumab had an overall safety profile comparable to placebo (Table 4). There were no cardiovascular adverse events in this subpopulation of patients.

**Table 4:** Incidence of adverse events during the double-blind treatment phase

	Placebo ***N*** = 83	Erenumab 70 mg ***N*** = 68	Erenumab 140 mg ***N*** = 81
All treatment-emergent adverse events, n (%)	56 (67.5)	42 (61.8)	42 (51.9)
Grade 3^a^	5 (6.0)	2 (2.9)	3 (3.7)
Serious	2 (2.4)	2 (2.9)	2 (2.5)
Leading to discontinuation of study drug	3 (3.6)	1 (1.5)	3 (3.7)
Fatal	0 (0.0)	0 (0.0)	0 (0.0)
Adverse events in ≥2% of patients, n (%)^b^			
Nasopharyngitis	10 (12.0)	8 (11.8)	8 (9.9)
Upper respiratory tract infection	2 (2.4)	6 (8.8)	5 (6.2)
Nausea	3 (3.6)	3 (4.4)	3 (3.7)
Influenza	1 (1.2)	2 (2.9)	3 (3.7)
Insomnia	0 (0.0)	0 (0.0)	3 (3.7)
Fatigue	1 (1.2)	2 (2.9)	2 (2.5)
Sinusitis	0 (0.0)	2 (2.9)	2 (2.5)
Vomiting	3 (3.6)	1 (1.5)	2 (2.5)
Injection site erythema	1 (1.2)	1 (1.5)	2 (2.5)
Urinary tract infection	5 (6.0)	2 (2.9)	1 (1.2)
Headache	0 (0.0)	2 (2.9)	1 (1.2)
Cardiovascular events, n (%)^c^	0 (0.0)	0 (0.0)	0 (0.0)

Adverse events were graded using CTCAE version 4.03. All serious adverse events were single-occurrence events

*Abbreviations*: *CTCAE* Common Terminology Criteria for Adverse Events

^a^There were no grade 4 adverse events

^b^In any of the treatment groups

^c^Based on the following search criteria: ischemic central nervous system vascular conditions, ischemic heart disease, and peripheral arterial disease

## Discussion

Consistent with the overall STRIVE population, preventive treatment with erenumab 70 mg and 140 mg vs placebo resulted in statistically significant improvements in MMD and acute MSMD and achievement of ≥ 50% response in this subpopulation of patients with a self-reported history of menstrual migraine. The overall incidence of treatment-emergent adverse events was also consistent with the overall STRIVE population.

Because of the frequency and burden of migraine in women with menstrual migraine, the majority qualify for preventive treatment [31]. However, although there are strategies for short-term prevention of menstrual migraine, limited options are available for long-term prevention [14]. It is, therefore, of interest that the efficacy and safety profiles of erenumab in this subgroup were similar to the overall episodic migraine population of STRIVE, in which erenumab significantly reduced the number of MMD and MSMD and increased the odds of achieving ≥ 50% reduction from baseline in MMD [29]. A subgroup analysis of MMD among women who received hormonal contraception suggests that exogenous hormones do not impact the efficacy of erenumab in this patient population; however, the sample sizes of these subgroups were too small to draw any definitive conclusions. Further investigation appears warranted, as several studies suggest that fluctuations of ovarian steroid hormone levels may modulate CGRP, with high estrogen states being related to an increase in CGRP levels in general, although the exact mechanistic interactions between ovarian steroid hormones and CGRP are not fully understood [32].

The prevalence of menstrual migraine depends on how it is defined and recorded, and there may be substantial differences in prevalence rates of menstrual migraine determined by self-report. For example, in population-based studies [11, 13], the reported prevalence of menstrual migraine is about 20% of women with migraine (approximately 7% of the general female population), compared with 11% of women with migraine when prospectively assessed in the context of clinic-based studies [33]. The prevalence of self-reported menstrual migraine determined in our analysis (28.5%) is higher than that observed in clinical trials that prospectively assessed menstrual migraine. Although our data on menstrual migraine were collected in the context of a prospective clinical trial, this finding may be due to self-reported data that were not confirmed with headache diaries during the study.

Our exploratory analysis is limited by our inability to differentiate PMM and MRM, and our inability to examine the effect of treatment on intermenstrual vs perimenstrual migraine days. Given that the study required at least 4 MMDs and the fact that 214 (92%) patients had a baseline MMD > 5, it is reasonable to conclude that the majority of women likely belonged to the MRM group, experiencing both menstrual and non-menstrual migraine attacks, although this information was not collected. The patients categorized as having menstrual migraine in our study have similar characteristics to “real-world” patients with menstrual migraine, who are generally identified based on retrospective self-report of perimenstrual migraine attacks during clinical encounters with the treating clinician rather than with prospective headache diaries. Similar to the general population [34], approximately one-third of patients with menstrual migraine reported that they were taking oral contraceptives/hormone therapy during the study, which may be an important confounder in terms of efficacy in this subgroup of patients. In addition, women who experience menstrual migraine may be more likely to use continuous contraceptive/hormonal options and may have been misclassified if they were not currently experiencing perimenstrual attacks. Furthermore, the relatively small sample size likely contributed to variability of effect and statistical significance at some time points. Variability was reduced, however, by analyzing the mean monthly efficacy over months 4–6, the primary prespecified analytic approach.

## Conclusions

In summary, these exploratory data from a large phase 3 study of erenumab in patients with menstrual migraine attacks (including both PMM and MRM subgroups) are consistent with the overall STRIVE episodic migraine population and support the efficacy of erenumab in this specific subgroup of women.

## Data Availability

Qualified researchers may request data from Amgen clinical studies. Complete details are available at http://www.amgen.com/datasharing.

## Abbreviations

CGRP: Calcitonin gene-related peptide
ICHD: The International Classification of Headache Disorders
MMD: Monthly migraine days
MRM: Menstrually related migraine
MSMD: Migraine-specific medication days
PMM: Pure menstrual migraine
